# Association between famine exposure in early life and risk of hospitalization for heart failure in adulthood

**DOI:** 10.3389/fpubh.2022.973753

**Published:** 2022-09-06

**Authors:** Chao-lei Chen, Jia-bin Wang, Yu-qing Huang, Ying-qing Feng

**Affiliations:** ^1^Department of Cardiology, Guangdong Cardiovascular Institute, Guangdong Provincial People's Hospital, Guangdong Academy of Medical Sciences, Guangzhou, China; ^2^Global Health Research Center, Guangdong Provincial People's Hospital, Guangdong Academy of Medical Sciences, Guangzhou, China

**Keywords:** Chinese famine, heart failure, economic status, hypertension, diabetes, dyslipidemia

## Abstract

**Background:**

Few studies have reported the association of early life exposure to famine with the risk of heart failure. The current study aimed to investigate whether exposure to famine in early life is associated with a higher risk of hospitalization for heart failure in adulthood.

**Methods:**

We used data from participants included in the sub-cohort of the China Patient-centered Evaluative Assessment of Cardiac Events Million Persons Project in Guangdong Province. Specific years of birth were used to define the famine-exposed group (born during the famine of 1959–1962), the pre-famine group (born before the famine [1954–1957], and the post-famine group (born after the famine [1964–1967]). Multivariable-adjusted generalized linear models were used to examine the associations of early life famine exposure with the risk of hospitalization for heart failure.

**Results:**

A total of 36,212 participants were enrolled in this analysis with a median age of 57.4 years and 37.5% of them were men. Compared with the post-famine group, famine births and pre-famine births were associated with increased risk of heart failure (OR: 1.96 [1.56–2.48] and OR: 1.62 [1.07–2.47], respectively). When compared with the age-balanced non-exposed group, the famine-exposed group was also significantly associated with increased risk of heart failure (OR: 1.32 [1.11–1.57]). The associations were stronger in participants with better economic status and in participants with hypertension, diabetes, and dyslipidemia (P for interaction < 0.05).

**Conclusion:**

Early life exposure to the Chinese famine is associated with an elevated risk of hospitalization for heart failure in adulthood.

## Introduction

Heart failure (HF) represents the advanced manifestation of various heart diseases and is one of the leading causes of mortality and disability around the world (1, 2). It is estimated that 64.3 and 8.9 million people are suffering from HF worldwide and in China, respectively (3, 4). Other than known risk factors such as high blood pressure, diabetes, and obesity, an increasing body of evidence, mostly from animal models, has shown the implications of exposure to poor nutrition early in life in the growth and developments of cardiometabolic outcomes (5–7). Although such studies in human beings is challenging to conduct, episodes of famine in recent history have provided some “natural” experimental settings to explore the role of undernutrition in early life in the development of cardiometabolic diseases in adulthood (8–10).

Previous famine studies have indicated significant associations of famine exposure with well-defined cardiometabolic risk factors such as obesity, hypertension, dyslipidemia, and diabetes, as well as cardiovascular diseases (CVD) including coronary heart disease, myocardial infarction, and stroke (11–18). However, the association between early life exposure to famine and risk of HF in adulthood has not been well-studied. Using sub-cohort of the China Patient-Centered Evaluative Assessment of Cardiac Events Million Persons Project (PEACE MPP) in Guangdong Province, we therefore investigated the associations of early life exposure to the Chinese famine of 1959–1962 with the risk of hospitalization for HF in adulthood.

## Methods

### Study population

**Figure 1:**
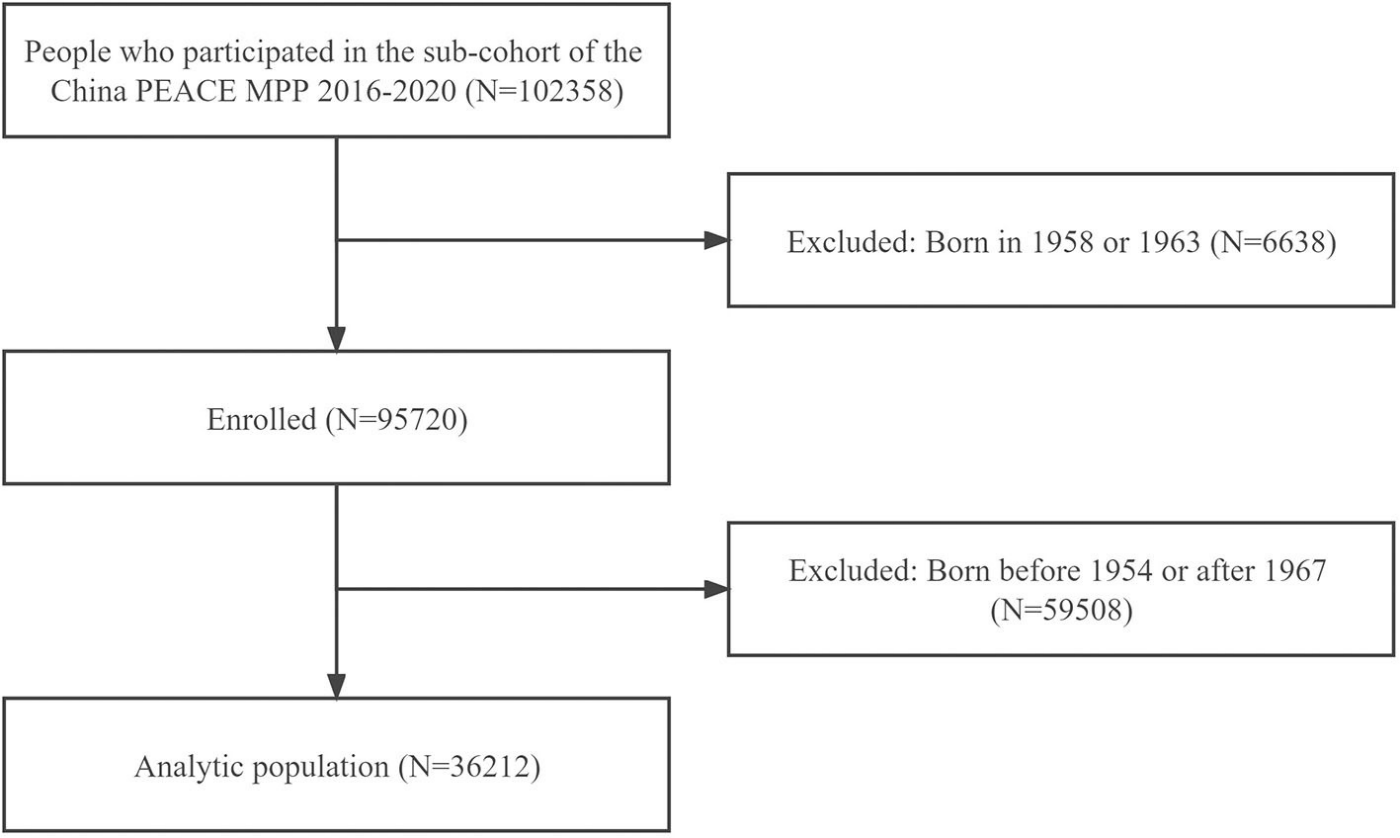
Flow chart of study participants. PEACE MPP, Patient-Centered Evaluative Assessment of Cardiac Events Million Persons Project.

The China PEACE MPP is a nationwide, government-funded, and population-based CVD screening study for identifying individuals with high CVD risk. The design and methods of China PEACE MPP have been described elsewhere (19–21). The current study was conducted in a sub-cohort of the China PEACE MPP, and 102358 participants were initially enrolled in 8 sites across Guangdong Province from 1 January 2016 to 31 December 2020. The inclusion criteria consisted of (1) registered in the local registration records, (2) community-dwelling residents who settled locally more than 6 months, and (3) aged 35 to 75 years. We excluded participants born in 1958 and 1963 from the analysis to minimize potential misclassification (*N* = 6,638), as previous studies have suggested (10). This is because the exact start or end date of Chinese famine is not clear according to nationally mortality rates around those years (22, 23). Among eligible participants, those who were born between 1954 and 1967 were included in the current study (*N* = 36,212) (Figure 1). We compared the characteristics of participants who were included in the present study or not and found no significant difference between them (all *P* > 0.05). This study was approved by both the Central Ethics Committee at the China National Center for Cardiovascular Disease and the Ethics Committee of Guangdong Provincial People's Hospital [No. GDREC2016438H (R2)]. Written informed consents were obtained from all participants.

### Famine definition

As one of the greatest famines in human history, the Chinese famine of 1959 to 1962 affected mainland China and caused ~30 million deaths (24). Consistent with previous Chinese famine studies (10, 25), participants were categorized into 3 groups using birth year as the proxy variable of exposure to famine: pre-famine group (born in 1954–1957), famine-exposed group (born in 1959–1962), and post-famine group (born in 1964–1967). The birth date for each participant was obtained from their resident identification card.

### Study outcomes

The main outcome of this study was hospitalization for HF. For both famine-exposed group and control group, participants' inpatient records from the Hospital Discharge Register System were reviewed and identified by trained staff who were blinded to the exposure status as well as other individual information. Events of hospitalization for HF were ascertained using code of I50 of the Tenth Revision of International Classification of Diseases (ICD-10) (26). All events were independently reviewed and verified by a panel of three experienced experts, including two cardiologists and one statistician.

### Assessment of covariates

We assessed covariates that included age, sex, education, occupation, economic status (annual income), marriage, smoking and drinking status, self-reported history of hypertension, diabetes, and dyslipidemia, and self-reported current use of antihypertensive, antidiabetic, lipid-lowering, and antiplatelet medications, and statin therapy. Smoking and drinking status were collected by asking the question “Do you currently smoke cigarettes or drink alcohol?” For each participant, physical examinations were also performed to measure systolic blood pressure (SBP), diastolic blood pressure (DBP), height, weight, and waist circumference. Blood pressure was measured twice on the right upper arm after 5 min of rest in a seated position using an electronic blood pressure monitor (Omron HEM-7430; Omron Corporation, Kyoto, Japan) and a standard protocol. Fasting blood glucose (FBG) was measured using fingertip blood samples (BeneCheck BK6–20M Multi-Monitoring System, Suzhou Pu Chun Tang Biotechnology, China). Lipid profile including triglyceride (TG), total cholesterol (TC), high-density lipoprotein cholesterol (HDL-C), and low-density lipoprotein cholesterol (LDL-C) were measured by a rapid lipid analyzer (CardioChek PA Analyzer; Polymer Technology Systems, Indianapolis, Indiana, USA). Body mass index (BMI) was calculated by dividing the weight in kilograms by the square of height in meters. Hypertension was determined based on self-reported using of antihypertensive drugs, or SBP ≥ 140 mmHg and/or DBP ≥ 90 mmHg (27). Dyslipidemia was determined based on self-reported using of lipid-lowering medications, or TC ≥ 6.2 mmol/L and/or LDL-C ≥ 4.1 mmol/L (28). Diabetes was determined based on self-reported using of antidiabetic drugs, or FBG ≥ 7.0 mmol/L (29).

### Statistical analysis

Continuous variables were described as median (interquartile range) for non-normal distribution. Categorial variables were described as number and percentage. We compared the characteristics of study participants according to famine exposure status using chi-squared test, Wilcoxon rank sum test, one-way ANOVA or Kruskal-Wallis H test as appropriate. Multivariable-adjusted generalized linear models were used to estimate the odds ratio (OR) and 95% confidence interval (CI) of HF for famine group and pre-famine group compared with post-famine group. Model 1 was adjusted for age and sex; Model 2 with additional adjustment for marital status, educational status, occupation, economic status, smoking, drinking, and BMI; Model 3 with additional adjustment for hypertension, diabetes, dyslipidemia, and current use of antiplatelet medications and statin therapy. We conducted a series of stratification analyses by sex (men vs. women), economic status (annual income <50,000 vs. ≥ 50,000 yuan), current smoking status (no vs. yes), hypertension (no vs. yes), diabetes (no vs. yes), dyslipidemia (no vs. yes), and BMI (<24 vs. ≥ 24 kg/m^2^). In the sensitivity analysis, to reduce the age gap between groups, we combined the pre-famine and post-famine groups together as an age-balanced non-exposed group to test the robustness of the main results, as suggested by previous studies (10, 30, 31). All analyses were conducted with R statistical software version 3.33 (R Project for Statistical Computing). *P* < 0.05 was considered significant.

## Results

### Characteristics of study participants

A total of 36,212 participants were included in the current study, of which 37.5% were men and the median age was 57.4 years. The prevalence of HF in the pre-famine group, famine group, and post-famine were 0.9, 2.0, and 2.8%, respectively. Compared with the pre-famine group, the famine group and post-famine group had higher prevalence of traditional CVD risk factors such as smoking, hypertension, diabetes, and dyslipidemia (Table 1). Of participants included, 669 (1.85%) had events of hospitalizations for HF, and those with HF were older and more likely to be men, had higher prevalence of cardiovascular risk factors such as smoking, BMI, hypertension, diabetes, and dyslipidemia, and had higher rate of current use of antihypertensive, antidiabetic, lipid-lowering, antiplatelet drugs, and statin therapy (Table 2).

**Table 1:** Characteristics of study participants according to famine exposure among 36,212 participants.

**Characteristics**	**Total**	**Pre-famine group**	**Famine group**	**Post-famine group**	***P*-value**
Number	36,212	12,542	10,493	13,177	
Age, y	57.4 (53.5–61.7)	52.6 (51.6–53.7)	57.2 (56.1–58.5)	62.5 (61.4–63.5)	<0.001
Men, *n* (%)	13,570 (37.5)	4,704 (37.5)	3,655 (34.8)	5,211 (39.5)	<0.001
Educational status (high school or above), *n* (%)	8,953 (24.7)	2,976 (23.7)	3,107 (29.6)	2,870 (21.8)	<0.001
Occupation (Farmer), *n* (%)	5,202 (14.4)	1,547 (12.3)	1,516 (14.4)	2,139 (16.2)	<0.001
Economic status (annual income ≥50,000 yuan), *n* (%)	15,899 (43.9)	5,695 (45.4)	4,574 (43.6)	5,630 (42.7)	<0.001
Marriage (married), *n* (%)	33,096 (91.4)	11,577 (92.3)	9,585 (91.3)	11,934 (90.6)	<0.001
Current smoker, *n* (%)	6,365 (17.6)	2,101 (16.8)	1,793 (17.1)	2,471 (18.8)	<0.001
Current drinker, *n* (%)	2,010 (5.6)	686 (5.5)	535 (5.1)	789 (6.0)	<0.001
BMI, kg/m^2^	24.1 (22.0–26.3)	24.2 (22.2–26.4)	24.1 (22.1–26.3)	23.9 (21.8–26.2)	<0.001
Waist circumference, cm	84.0 (78.0–90.0)	84.0 (78.0–90.0)	84.0 (78.0–90.0)	85.0 (78.0–90.9)	<0.001
SBP, mm Hg	131.0 (119.5–143.5)	127.5 (117.0–140.0)	131.0 (119.5–144.0)	133.5 (122.0–147.0)	<0.001
DBP, mm Hg	80.0 (72.5–87.5)	79.5 (72.5–87.5)	80.0 (72.5–87.5)	80.0 (72.5–87.0)	0.596
FBG, mg/dL	102.6 (91.8–115.2)	100.8 (91.8–113.4)	102.6 (91.8–115.2)	102.6 (91.8–115.2)	<0.001
TG, mg/dL	122.1 (90.3–176.1)	120.5 (88.6–172.8)	124.9 (92.1–179.9)	123.2 (92.1–177.2)	<0.001
TC, mg/dL	191.8 (162.5–223.9)	189.6 (161.0–220.2)	194.7 (164.9–227.2)	192.7 (162.9–226.0)	<0.001
LDL–C, mg/dL	106.2 (82.2–133.2)	104.5 (81.3–130.0)	108.7 (84.0–137.0)	106.8 (82.4–135.1)	<0.001
HDL-C, mg/dL	54.8 (44.8–67.6)	55.0 (44.5–67.3)	55.0 (44.9–67.7)	55.7 (45.3–68.1)	<0.001
Hypertension, *n* (%)	16,385 (45.2)	4,791 (38.2)	4,796 (45.7)	6,798 (51.6)	<0.001
Diabetes, *n* (%)	6,699 (18.5)	2,025 (16.1)	2,083 (19.9)	2,591 (19.7)	<0.001
Dyslipidemia, *n* (%)	8,033 (22.2)	2,349 (18.7)	2,561 (24.4)	3,123 (23.7)	<0.001
Current use of antihypertensive drugs, *n* (%)	7,942 (21.9)	2,120 (16.9)	2,324 (22.1)	3,498 (26.5)	<0.001
Current use of antidiabetic drugs, *n* (%)	2,965 (8.2)	764 (6.1)	962 (9.2)	1,239 (9.4)	<0.001
Current use of lipid-lowering drugs, *n* (%)	1,614 (4.5)	396 (3.2)	504 (4.8)	714 (5.4)	<0.001
Current use of statin therapy, *n* (%)	275 (0.8)	59 (0.5)	90 (0.9)	126 (1.0)	<0.001
Current use of antiplatelet drugs, *n* (%)	197 (0.5)	41 (0.3)	47 (0.4)	109 (0.8)	<0.001

Data are presented as median (IQR) for non-normally distributed variables, and numbers (percentages) for categorical variables. BMI, body mass index; SBP, systolic blood pressure; DBP, diastolic blood pressure; FBG, fasting blood glucose; TC, total cholesterol; TG, triglyceride; LDL-C, low-density lipoprotein cholesterol; HDL-C, high-density lipoprotein cholesterol.

**Table 2:** Characteristics of study participants with or without hospitalization for heart failure among 36,121 participants.

**Characteristics**	**Total**	**Without HF**	**With HF**	***P*-value**
Number	36,212	35,543	669	
Age, y	57.4 (53.5–61.7)	57.3 (53.5–61.7)	60.4 (56.5–62.7)	<0.001
Men, *n* (%)	13,570 (37.5)	13,182 (37.1)	388 (58.0)	<0.001
Educational status (high school or above), *n* (%)	8,953 (24.7)	8,783 (24.7)	170 (25.4)	0.71
Occupation (Farmer), *n* (%)	5,202 (14.4)	5,102 (14.4)	100 (14.9)	0.71
Economic status (annual income ≥ 50,000 yuan), *n* (%)	15,899 (43.9)	15,605 (43.9)	294 (43.9)	0.99
Marriage (married), *n* (%)	33,096 (91.4)	32,487 (91.4)	609 (91.0)	0.79
Current smoker, *n* (%)	6,365 (17.6)	6,170 (17.4)	195 (29.1)	<0.001
Current drinker, *n* (%)	2,010 (5.6)	1,962 (5.5)	48 (7.2)	0.08
BMI, kg/m2	24.1 (22.0–26.3)	24.1 (22.0–26.3)	24.9 (22.6–27.2)	<0.001
Waist circumference, cm	84.0 (78.0–90.0)	84.0 (78.0–90.0)	88.0 (80.0–94.0)	<0.001
SBP, mm Hg	131.0 (119.5–143.5)	131.0 (119.5–143.5)	135.0 (122.0–151.5)	<0.001
DBP, mm Hg	80.0 (72.5–87.5)	80.0 (72.5–87.5)	81.0 (73.0–89.0)	0.009
FBG, mg/dL	102.6 (91.8–115.2)	101.2 (91.8–115.2)	106.2 (91.8–127.8)	<0.001
TG, mg/dL	122.4 (90.5–176.5)	122.4 (90.5–176.5)	128.6 (94.0–190.7)	0.008
TC, mg/dL	192.3 (162.9–224.5)	192.3 (163.3–224.5)	174.5 (142.8–214.4)	<0.001
LDL-C, mg/dL	109.9 (84.4–131.2)	110.3 (84.4–131.6)	98.7 (72.4–122.3)	<0.001
HDL-C, mg/dL	55.0 (44.9–67.7)	55.3 (44.9–67.7)	49.5 (40.6–61.9)	<0.001
Hypertension, *n* (%)	16,385 (45.2)	15,950 (44.9)	435 (65.0)	<0.001
Diabetes, *n* (%)	6,699 (18.5)	6,469 (18.2)	230 (34.4)	<0.001
Dyslipidemia, *n* (%)	8,033 (22.2)	7,838 (22.1)	195 (29.1)	0.009
Current use of antihypertensive drugs, *n* (%)	7,942 (21.9)	7,639 (21.5)	303 (45.3)	<0.001
Current use of antidiabetic drugs, *n* (%)	2,965 (8.2)	2,829 (8.0)	136 (20.3)	<0.001
Current use of lipid-lowering drugs, *n* (%)	1,614 (4.5)	1,512 (4.3)	102 (15.2)	0.009
Current use of statin therapy, *n* (%)	275 (0.8)	255 (0.7)	20 (3.0)	<0.001
Current use of antiplatelet drugs, *n* (%)	197 (0.5)	178 (0.5)	19 (2.8)	<0.001

Data are presented as median (IQR) for non-normally distributed variables, and numbers (percentages) for categorical variables.

PEACE MPP, Patient-Centered Evaluative Assessment of Cardiac Events Million Persons Project; HF, heart failure; BMI, body mass index; SBP, systolic blood pressure; DBP, diastolic blood pressure; FBG, fasting blood glucose; TC, total cholesterol; TG, triglyceride; LDL-C, low-density lipoprotein cholesterol; HDL-C, high-density lipoprotein cholesterol.

### Famine exposure and HF

Both the famine group and pre-famine group were associated with increased risk of hospitalization for HF compared with the post-famine group (OR = 1.96 for famine births, 95% CI: 1.56–2.48, and OR = 1.62 for pre-famine births, 95% CI: 1.07–2.47), after adjusting for age, sex, marriage, educational status, occupation, family annual income, smoking, drinking, BMI, hypertension, diabetes, dyslipidemia, statin therapy, and current use of antiplatelet drugs (Table 3). In the sensitivity analysis, famine group was also associated with elevated risk of HF compared with the age-balanced non-exposed group (multivariable-adjusted OR = 1.32, 95% CI: 1.11–1.57) (Supplementary Table 1).

**Table 3:** Odds ratio with 95% CI of hospitalization for heart failure according to famine exposure among 36,212 participants.

	**Post-famine group**	**Famine group**	**Pre-famine group**
Case (%)	118 (0.9)	211 (2.0)	340 (2.6)
Model 1	1.0	2.13 (1.70–2.69)	1.70 (1.12–2.59)
Model 2	1.0	2.14 (1.70–2.70)	1.68 (1.11–2.56)
Model 3	1.0	1.96 (1.56–2.48)	1.62 (1.07–2.47)

Model 1: adjusted for age and sex.

Model 2: adjusted for age, sex, smoking, drinking, marriage, educational status, occupation, economic status, and body mass index.

Model 3: adjusted for age, sex, smoking, drinking, marriage, educational status, occupation, economic status, body mass index, hypertension, diabetes, dyslipidemia, statin therapy, and current use of antiplatelet drugs.

### Stratification analyses

**Figure 2:**
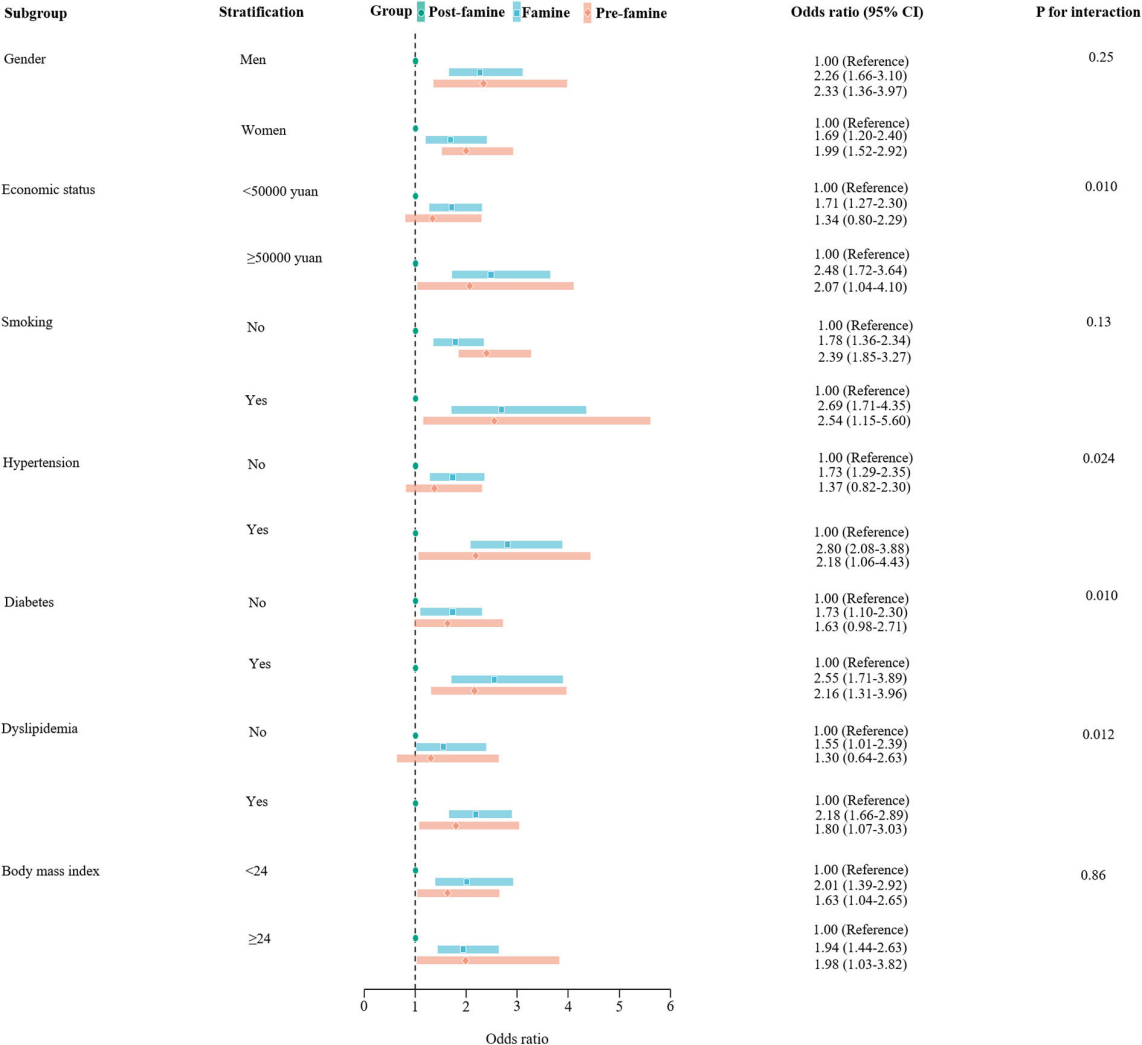
Stratification analysis of associations of pre-famine and famine births with risk of hospitalization for heart failure compared with post-famine births. Presented were multivariable-adjusted generalized linear models with adjustment for age, sex, marriage, educational status, occupation, economic, smoking, drinking, body mass index, hypertension, diabetes, dyslipidemia, current use of antiplatelet medications, and statin therapy. The square in the middle represents the odds ratio of the risk estimation, and the bar represents its 95% CI.

Stratification analyses by sex, smoking status, and BMI did not show significant differences in the associations of early life exposure to the Chinese famine with hospitalization for HF in those subgroups. However, stratification analyses by economic status showed that, compared with the post-famine group, the associations between famine group and risk of HF were stronger in those with income ≥50,000 yuan per year than in those with income <50,000 yuan (OR_≥50, 000*yuan*_: 2.48 [1.72–3.64] vs. OR _ <50, 000yuan_ 1.71 [1.27–2.30], P for interaction = 0.010). The associations of famine group with HF compared with the post-famine group were also stronger in those with hypertension (OR: 2.80 [2.08–3.88] in hypertensive participants vs. 1.73 [1.29–2.35] in non-hypertensive participants, P for interaction = 0.024), in those with diabetes (OR: 2.55 [1.71–3.89] in diabetic participants vs. 1.73 [1.10–2.30] in non-diabetic participants, P for interaction = 0.010), and in those with dyslipidemia (OR: 2.18 [1.66–2.89] in participants with dyslipidemia vs. 1.55 [1.01–2.39] in participants without dyslipidemia, P for interaction = 0.012) (Figure 2). The results were similar for the pre-famine group when comparing to the post-famine group and remained robust when using the age-balanced control group as reference (Supplementary Figure 1).

## Discussion

Our large population-based study revealed that early life exposure to the Chinese famine of 1959–1962 was significantly associated with increased risk of hospitalization for HF in adulthood.

Although the associations between famine exposure early in life and self-reported cardiovascular diseases have been recognized in previous studies, evidence about the associations with rarely reported cardiometabolic outcomes such as hospitalization for HF is still lacking (15, 17, 32). Among 5,772 participants in the China Health and Retirement Longitudinal Study (CHARLS), Shi and colleagues (33) observed that early life exposure to the Chinese famine increased the risk of self-reported composite CVD events (OR = 2.87, 95% CI: 1.16–7.07). In the REACTION (Risk Evaluation of Cancers in Chinese Diabetic Individuals) study of 259,657 community-dwelling adults, Du et al. (17) showed that early life famine exposure was associated with higher risk of self-reported total CVD, coronary heart disease, myocardial infarction, and stroke. Similarly, among 92 284 participants from the China Kadoorie Biobank, Meng et al. (15) found that early life exposure to the Chinese famine was associated with increased risks of ischemic heart disease, cerebrovascular disease, and ischemic stroke, which were defined using local disease national health insurance system and ICD-10 codes. Our study including over 35,000 participants added the evidence on the positive relation between famine exposure early in life and risk of hospitalization for HF, which is the final stage of various kinds of CVD (2).

Inconsistent with previous studies that revealed sex differences in the associations between famine exposure and cardiometabolic outcomes such high blood pressure (10, 34), diabetes (35, 36), and self-reported CVD events (17), we found similar association between famine exposure to the Chinese famine and HF risk in men and women. The study design, participant selection, and definitions of exposed and non-exposed groups may contribute to these reported inconsistent findings. However, we found in the current study that participants with better economic status had increased risk of HF when exposed to famine in early life. One explanation is that socioeconomic status plays an important role in developing CVD, and it even had bigger effect than healthy lifestyles in adulthood (37). Indeed, Wang et al. (38) found that early-life famine exposure was positively associated with hyperuricemia in subjects with high economic status rather than in those with low economic status. These findings conformed the Barker hypothesis (39), which demonstrated that if the utero development of a thrifty phenotype mismatched the later plentiful environment, infants suffering from undernutrition will be more prone to cardiometabolic disease in later life. Additionally, we found that hypertensive and diabetic participants who were exposed to famine in early life had higher risk of HF than non-hypertensive participants. These findings were consistent with results from two representative cohorts of Chinese adults that provided evidence that early exposure to the Chinese famine of 1959–1962 exacerbated the association of hypertension, diabetes, and risk of CVD in later life (33, 40). In addition, we found that in the present study, dyslipidemia modified the effect of early life exposure to famine on later risk of HF, which was inconsistent with the REACTION study that found non-significantly stronger association between early life famine exposure and CVD risk in participant without dyslipidemia (17). This difference could be explained to the complex mediation of metabolic syndrome on the relationship between famine and CVD and the clustering of multimorbidity (17).

Although not well-understood yet, several plausible mechanisms can be responsible for the adverse associations of famine exposure early in life with HF risk in adulthood. First, animal experiments have revealed that prenatal and postnatal malnutrition can elevate blood pressure by altering the renin-angiotensin system (41, 42), and increase blood glucose by destroying pancreatic β-cell function (43, 44), which can then increase cardiovascular risk. Second, nutritional deficiency early in life can result in limited development in multiple organs and tissues, such as pancreas, adipose, and kidney, and then increased risk of cardiac diseases later in life (45, 46). Third, it has been reported that fetal exposure to famine was associated with changes in DNA methylation of genes involved in inflammation, adipogenesis, and glycolysis (47–49), therefore, epigenetic modifications may be a plausible mechanism linking famine exposure and cardiovascular health.

Several limitations should be kept in mind when interpreting our findings. First. although the birth date was commonly used to define famine exposure in this research field, this method may lead to misclassification bias and underestimated associations. Second, residual confounding was still likely given the nature of observational study design. Third, data relevant to dietary patterns, physical activity, and birth weight was not collected and thus cannot be adjusted in our analysis. Finally, we were unable to distinguish either subgroups of HF (i.e., HF with reduced or preserved ejection fraction) or causes of HF, and then unable to further explore the relationships between early life famine exposure and specific HF outcomes.

## Conclusion

Taken together, our study revealed that early life exposure to the Chinese famine of 1959–1962 was associated with increased risk of hospitalization for HF in adulthood. These associations were stronger in those with better economic status and those with hypertension, diabetes, or dyslipidemia.

## Data availability statement

The original contributions presented in the study are included in the article/Supplementary material, further inquiries can be directed to the corresponding author/s.

## Ethics statement

This study was approved by both the Central Ethics Committee at the China National Center for Cardiovascular Disease and the Ethics Committee of Guangdong Provincial People's Hospital [No. GDREC2016438H (R2)]. The patients/participants provided their written informed consent to participate in this study.

## Author contributions

C-lC and Y-qF: conceptualization and study design. C-lC, J-bW, and Y-qF: paper preparation. C-lC and J-bW: statistical analysis and data interpretation. All authors: investigation and reviewed and approved this manuscript.

## Funding

This work was supported by the Ministry of Finance of China and National Health and Family Planning Commission of China, the Key Area R&D Program of Guangdong Province (No.2019B020227005), the Climbing Plan of Guangdong Provincial People's Hospital (DFJH2020022), and Guangdong Provincial Clinical Research Center for Cardiovascular disease (2020B1111170011).

## Conflict of interest

The authors declare that the research was conducted in the absence of any commercial or financial relationships that could be construed as a potential conflict of interest.

## Publisher's note

All claims expressed in this article are solely those of the authors and do not necessarily represent those of their affiliated organizations, or those of the publisher, the editors and the reviewers. Any product that may be evaluated in this article, or claim that may be made by its manufacturer, is not guaranteed or endorsed by the publisher.
