# Supplementary material for: Association between famine exposure in early life and risk of hospitalization for heart failure in adulthood
Source: Front Public Health. 2022 Sep 6;10:973753. doi: 10.3389/fpubh.2022.973753 (PMC9485593; doi:10.3389/fpubh.2022.973753)
Supplement: Supplementary file 1 [file Data_Sheet_1.docx]

**Supplementary Table S1.** **Odds Ratio with 95% CI of Hospitalization for Heart Failure According to Early Life Exposure to the Chinese Famine Using Age-balanced Control**

|  | **Non-exposed** | **Exposed** | ***P* value** |
| --- | --- | --- | --- |
| Case (%) | 458 (1.8) | 211 (2.0) |  |
| Model 1 | Ref | 1.37 (1.15-1.63) | < 0.001 |
| Model 2 | Ref | 1.36 (1.15-1.62) | < 0.001 |
| Model 3 | Ref | 1.32 (1.11-1.57) | 0.002 |

Model 1: adjusted for age and sex.

Model 2: adjusted for age, sex, smoking, drinking, marriage, educational status, occupation, economic status, and body mass index.

Model 3: adjusted for age, sex, smoking, drinking, marriage, educational status, occupation, economic status, body mass index, hypertension, diabetes, dyslipidemia, statin therapy, and current use of antiplatelet drugs.


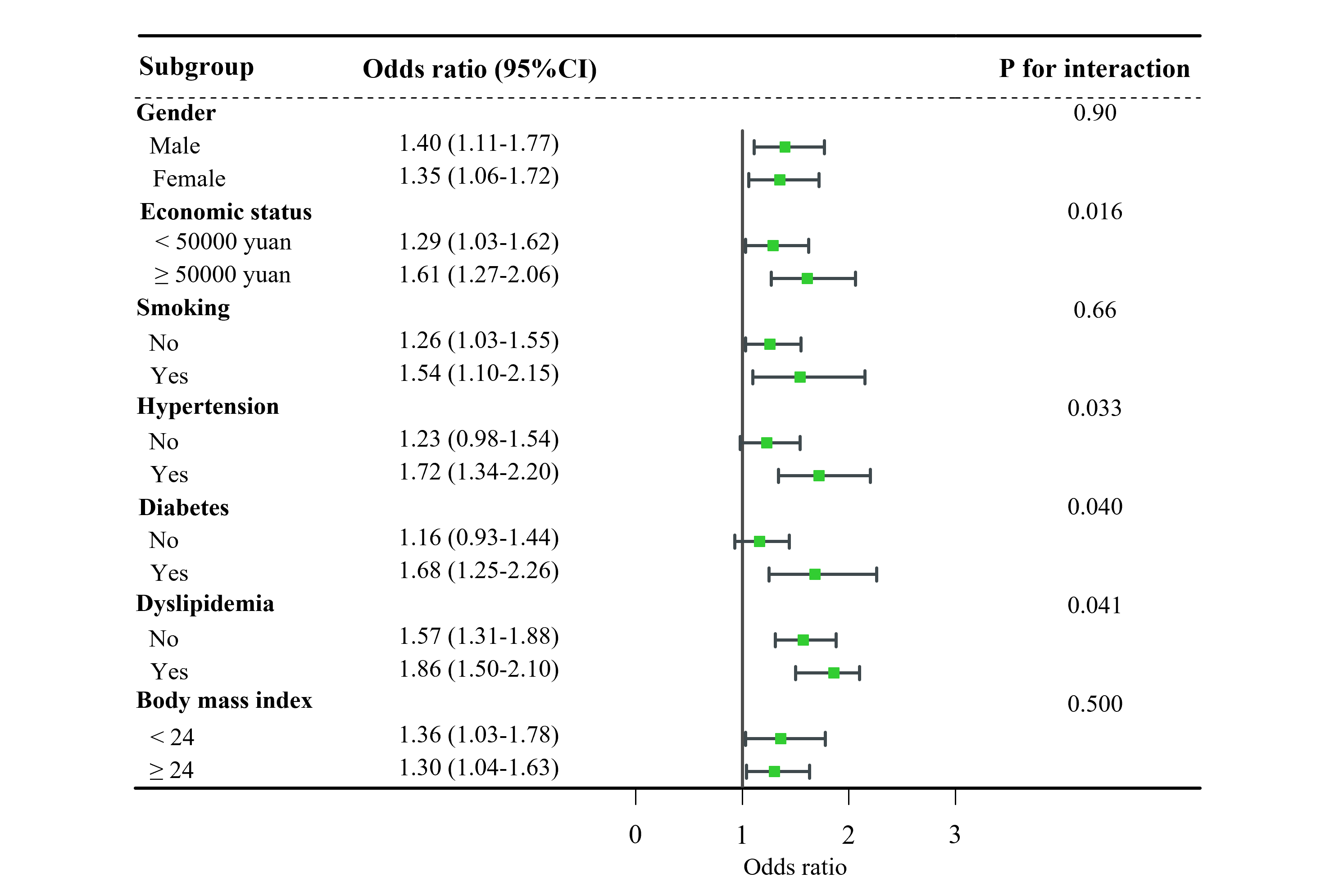


**Supplementary Figure S1. Stratification Analysis of Associations of Famine Exposure with Risk of Hospitalization for Heart Failure Using Age-balanced Control**

Presented were multivariable-adjusted generalized linear models with adjustment for age, sex, marriage, educational status, occupation, economic, smoking, drinking, body mass index, hypertension, diabetes, dyslipidemia, current use of antiplatelet medications, and statin therapy. The square in the middle represents the odds ratio of the risk estimation, and the bar represents its 95% CI.
